# The Impact of Insulin Resistance on Lung Volume Through Right Ventricular Dysfunction in Diabetic Patients—Literature Review

**DOI:** 10.3390/jpm15080336

**Published:** 2025-08-01

**Authors:** Daniel Radu, Oana-Andreea Parlițeanu, Andra-Elena Nica, Cristiana Voineag, Octavian-Sabin Alexe, Alexandra Maria Cristea, Livia Georgescu, Roxana Maria Nemeș, Andreea Taisia Tiron, Alexandra Floriana Nemeș

**Affiliations:** 1 Department of Emergency, Ilfov County Emergency Clinical Hospital, 022104 Bucharest, Romania; radudaniel301979@gmail.com; 2 Department of Diabetes, ‘Marius Nasta’ Institute of Pneumology, 050159 Bucharest, Romania; 3 Department of Diabetes, ‘Carol Davila’ University of Medicine and Pharmacy, 050474 Bucharest, Romania; dr.andranica@yahoo.com; 4 Department of Diabetes, Universitatea Dunărea de Jos, 800201 Galați, Romania; voineag.cristiana@gmail.com (C.V.); octavsabin@yahoo.com (O.-S.A.); 5 Department of Pneumology, ‘Marius Nasta’ National Institute of Pneumology, 050159 Bucharest, Romania; alexandra.maria.sora@gmail.com (A.M.C.); livialuculescu@gmail.com (L.G.);; 6 Faculty of Medicine, Titu Maiorescu University, 031592 Bucharest, Romania; 7 Department of Cardiology, ‘Carol Davila’ University of Medicine and Pharmacy, 050474 Bucharest, Romania; andreea.tiron@umfcd.ro; 8 Department of Neonatology, ‘Carol Davila’ University of Medicine and Pharmacy, 050474 Bucharest, Romania; alexandra-floriana.nemes@drd.umfcd.ro

**Keywords:** insulin resistance, type 2 diabetes, right ventricular dysfunction, lung volume

## Abstract

Insulin resistance (IR), a core component in the development of type 2 diabetes mellitus (T2DM), is increasingly recognized for its role in cardiovascular and pulmonary complications. This review explores the relationship between IR, right ventricular dysfunction (RVD), and decreased lung volume in patients with T2DM. Emerging evidence suggests that IR contributes to early structural and functional alterations in the right ventricle, independent of overt cardiovascular disease. The mechanisms involved include oxidative stress, inflammation, dyslipidemia, and obesity—factors commonly found in metabolic syndrome and T2DM. These pathophysiological changes compromise right ventricular contractility, leading to reduced pulmonary perfusion and respiratory capacity. RVD has been associated with chronic lung disease, pulmonary hypertension, and obstructive sleep apnea, all of which are prevalent in the diabetic population. As RVD progresses, it can result in impaired gas exchange, interstitial pulmonary edema, and exercise intolerance—highlighting the importance of early recognition and management. Therapeutic strategies should aim to improve insulin sensitivity and cardiac function through lifestyle interventions, pharmacological agents such as SGLT2 inhibitors and GLP-1/GIP analogs, and routine cardiac monitoring. These approaches may help slow the progression of RVD and its respiratory consequences. Considering the global burden of diabetes and obesity, and the growing incidence of related complications, further research is warranted to clarify the mechanisms linking IR, RVD, and respiratory dysfunction. Understanding this triad will be crucial for developing targeted interventions that improve outcomes and quality of life in affected patients.

## 1. Introduction

Insulin resistance (IR) has been proven to be the central element involved in the pathophysiology of type 2 diabetes mellitus (T2DM), later playing a crucial role in the development of microvascular complications, including the onset of right ventricular dysfunction (RVD) [1,2,3]. This dysfunction has been implicated in the reduction of lung volume in diabetic patients [4,5]. Understanding the pathophysiological mechanisms by which IR leads to RVD, and subsequently how this dysfunction impacts lung volume, is extremely important for managing the treatment strategies of patients with diabetes. Therefore, through this review, we aim to explore and better understand the mechanisms underlying these associations.

We decided to conduct a more thorough investigation of the aforementioned issue through a specialized literature review using the PubMed database, focusing on articles and meta-analyses that address the topics mentioned above.

## 2. Insulin Resistance and Type 2 Diabetes Mellitus

The central element implicated in the development of type 2 diabetes mellitus (T2DM) is insulin resistance (IR) associated with obesity, which is also linked to a relative insulin deficiency encountered later on in the evolution of T2DM [1]. Multiple recent studies incriminate the pathophysiological mechanisms created by IR as the main cause of T2DM and its chronic microvascular complications [2]. Obesity—particularly abdominal and visceral obesity—induces a higher degree of IR, and the mechanism believed to be responsible is the blockade of insulin receptors caused by the increased number of fat cells that derive from adipocytes [1].

In recent years, research has identified several chemical messengers specific to adipocytes, known as adipocytokines (such as tumor necrosis factor-alpha, adiponectin, and resistin), which also play an important role in the onset of IR, thus demonstrating that IR is, in fact, a condition created by cellular oxidative stress.

An unhealthy lifestyle that promotes the intake of ultra-processed foods high in carbohydrates and fats will lead to weight gain, which in turn induces obesity. Through the characteristic IR, patients will then develop disturbances in glucose homeostasis, ultimately leading to the development of type 2 diabetes mellitus [1,2].

To better understand the correlation between abdominal obesity and insulin resistance, over time, researchers have also investigated the genetic components that may be involved in this association [3]. It has been shown that a number of genes are implicated in metabolic syndrome and, therefore, in IR. These include the beta-3 adrenergic receptor gene, lipoprotein lipase, hormone-sensitive lipase, the peroxisome proliferator-activated receptor gamma (PPAR-γ), insulin receptor substrate-1 (IRS-1), and glycogen synthase. The discovery of these genes led to the conclusion that the onset of T2DM can be attributed to an interrelationship between genetic predisposition and environmental factors such as diet. With this knowledge, future treatment directions should aim to address both the intrinsic problems that cause insulin deficiency and the effects of insulin resistance [3].

Over time, clinical studies have demonstrated that insulin resistance (IR) is not only a triggering factor for type 2 diabetes mellitus (T2DM), but also a risk factor involved in cardiac remodeling. This is due to the effects of insulin resistance on lipid metabolism, as well as its role in oxidative stress and chronic inflammation.

Goldstein et al. stated that IR represents the central defect in the pathophysiology of T2DM; however, they did not extrapolate the effects of insulin resistance to other organs [1]. On the other hand, in the study conducted by Szoke and Gerich, the authors hypothesized that IR occurs even before the deterioration of insulin secretion, being an integral part of a broader metabolic dysfunction that also includes early cardiac involvement [2].

Another study, led by Groop, showed that IR has a genetically predetermined predisposition through the involvement of IRS-1, PPAR-γ, and β3-adrenergic receptor genes, which directly affect cellular insulin sensitivity [3]. Unfortunately, the findings of this study are based solely on population and genetic analyses and do not elucidate the direct mechanisms involved in right ventricular remodeling. For this reason, further studies are needed to investigate in more depth the expression of the aforementioned genes specifically in right ventricular myocardial tissue.

## 3. The Impact of Insulin Resistance on Right Ventricular Function

The effects of insulin resistance (IR) on the left ventricle have been well documented in the medical literature, but very few studies have addressed the impact of IR on the right ventricle (RV) [4]. One such study showed through linear regression, without ambiguity, that IR is associated with reduced systolic and diastolic function of the right ventricle. The results indicated that patients with IR have an increased risk of early right ventricular dysfunction, even in the absence of existing cardiovascular disease [4].

Insulin resistance and oxidative stress play a key role in the development of both left and right ventricular dysfunction, and by extension, in dilated cardiomyopathy (DCM) [5]. Although the pathophysiological mechanism involved in ventricular dysfunction appears to be similar for both ventricles, it is important to remember that the right ventricle has a distinct embryological origin and cellular composition compared to the left. The RV also has different biomechanical properties and is coupled to the pulmonary vascular bed, which has lower impedance [5].

Another potential association involved in the development of right ventricular dysfunction is the combination of T2DM and arterial hypertension, both of which can lead to heart failure (HF) with reduced ejection fraction [6]. In patients with HF, the presence of T2DM has been shown to be a risk factor for the onset of right ventricular dysfunction. Apparently, the right ventricle is directly affected by diabetes mellitus [6]. Starting from the premise that IR is the primary cause of T2DM, and that T2DM directly affects the RV leading to its dysfunction, we can extrapolate and state that IR is a risk factor for right ventricular dysfunction.

Another characteristic of patients with T2DM is the frequent coexistence of lipid metabolism disorders; diabetic patients are often also dyslipidemic [7]. Recent publications have shown a close relationship between inflammation and lipid profile, highlighting the need to reduce oxidative stress to lower the overall cardiovascular risk in these patients [8].

Considering the previously mentioned relationships between IR–oxidative stress and right ventricular dysfunction (RVD), and the oxidative stress–dyslipidemia link, one may also question whether there is a direct relationship between RVD and an altered lipid profile. Elevated total cholesterol and triglyceride levels have been associated with right ventricular dysfunction in pulmonary hypertension [9]. The presence of dyslipidemia has been shown to worsen the prognosis in patients with pulmonary hypertension, significantly reducing the right ventricular ejection fraction.

The effects of IR on lipid metabolism are also well known. It has been documented that elevated triglyceride levels, low levels of HDL cholesterol (high-density lipoprotein cholesterol), and high levels of LDL cholesterol (low-density lipoprotein cholesterol) are essential components of metabolic syndrome and, implicitly, of IR [10].

The relationship between oxidative stress, obesity, and insulin resistance has previously been established as a central component in the development of metabolic syndrome and its complications [1]. Mortality in patients with RVD has been shown to be directly proportional to body mass index (BMI); in other words, obesity is a significant risk factor in patients with right ventricular impairment [11].

As a brief conclusion to this section, based on the points mentioned above, we can affirm that there is a clear link between right ventricular dysfunction and insulin resistance through the already demonstrated relationships between RVD and obesity, oxidative stress, dyslipidemia, and diabetes mellitus—all of which contribute to ventricular remodeling and/or reduced ejection fraction, thus impairing ventricular function.

Brittain et al. described another mechanism involved in the impairment caused by insulin resistance to the right ventricle: they stated that RV failure can be induced by elevated levels of fatty acids from myocardium, thus lipotoxicity is a cause of RVD in patients with pulmonary arterial hypertension [12].

Another research that clearly states the relationship between IR and RDV in patients with pulmonary arterial hypertension is the one of Hemnes et al. [13]. Insulin resistance was described to be highly prevalent in patients with pulmonary arterial hypertension, especially in the group of patients with the highest mortality [13].

In a study conducted by Min and collaborators in 2022, a link between insulin resistance (IR) and right ventricular diastolic dysfunction was highlighted; this association was demonstrated independently of other coexisting cardiovascular diseases in the patients [4]. This was an observational study that described a linear regression analysis applied to a general population sample; however, due to its observational nature, the study cannot establish causality between the two pathologies.

In the same context, Hemnes et al. (2019) showed that IR is extremely prevalent among patients with pulmonary arterial hypertension, particularly in those with a high mortality rate. In their study, Hemnes and colleagues suggested that there is a direct pathophysiological link between IR and right ventricular remodeling [13].

## 4. The Relationship Between Right Ventricular Dysfunction and Lung Volume

The right ventricle plays an essential role in pumping blood to the lungs for oxygenation. Its dysfunction can lead to systemic venous congestion and inadequate pulmonary perfusion. Right ventricular dysfunction (RVD) is commonly associated with chronic pulmonary diseases; in these conditions, chronic hypoxemia and destruction of the pulmonary vascular bed result in increased right ventricular afterload [14]. A hallmark of RVD is right ventricular hypertrophy with preserved myocardial contractility and preserved ejection fraction. In chronic lung diseases characterized by prolonged hypoxemia, right ventricular hypertrophy is frequently observed. In cases of acute exacerbations of chronic pulmonary pathologies, right ventricular failure can also occur, although this is relatively rare [14,15].

Another association is with obstructive sleep apnea syndrome (OSA): 71% of patients with OSA presented with RVD at echocardiography [14]. It is unclear what the mechanism behind this association is, be it the result of OSA or derived from other comorbidities.

The most frequent association is between right ventricular dysfunction and pulmonary arterial hypertension [16].

From a pathophysiological perspective, the mechanisms underlying RVD may include pressure overload, volume overload, and myocardial disease—mechanisms that often occur simultaneously [17]. Additional contributing factors may include diastolic dysfunction and ventricular interdependence [17].

Possible causes of RVD include right ventricular outflow tract obstruction (e.g., unrepaired Tetralogy of Fallot), pulmonary stenosis (valvular, infundibular, supravalvular, or peripheral branch level), constrictive pericarditis, and chronic pulmonary hypertension [17].

The consequences of RVD include reduced gas diffusion capacity and decreased pulmonary compliance, which translate into a reduction in lung volume and exercise capacity. Moreover, there is a theoretical risk that venous congestion can lead to interstitial pulmonary edema, impairing gas exchange and contributing to respiratory symptoms such as dyspnea. Thus, RVD induced by insulin resistance in patients with type 2 diabetes has a direct impact on respiratory function [14].

Right ventricular function is negatively affected in patients with pulmonary arterial hypertension, due to the activation of the inflammation/fibrosis axis; this axis contributes to elevated levels of IL-6 (Interleukin 6) in these patients [18,19]. In these patients, who were found to have elevated IL-6 levels, an increase in NT-proBNP (N-terminal pro b-type natriuretic peptide) values was also observed [20]. Elevated IL-6 transcription levels have been detected in the right ventricular tissue of patients with pulmonary arterial hypertension and decompensated right ventricular dysfunction. When comparing patients with compensated RV dysfunction to those with decompensated RV dysfunction, the latter showed the most enriched pathway to be the Janus kinase/signal transducers and activators of the transcription (JAK/STAT) signaling pathway, which is activated by IL-6 [21].

It has been demonstrated that in patients with advanced right ventricular dysfunction, documented through imaging and hemodynamic function, multiple inflammatory and extracellular matrix remodeling pathways are upregulated. This finding has been confirmed in both mice and humans [22]. In a study led by Boucherat et al., fibrotic serum markers were shown to be good predictors of right ventricular dysfunction, with the pro-fibrotic latent transforming growth factor-β binding protein 2 (LTBP-2) also identified as a predictor of right ventricular failure. The severity of RV dysfunction is characterized by elevated levels of this aforementioned protein [21].

It has been demonstrated that in patients with pulmonary arterial hypertension (PAH) and low cardiac reserve, physical exertion leads to increased right-sided cardiac pressure and right ventricular stiffness, as well as decreased left ventricular filling and stroke volume, compared to PAH patients with high cardiac reserve [23]. Right ventricular stiffness leads to pulmonary artery stiffness, and, as a consequence of this mechanism, lung volume decreases.

For the first time, Cubero Salazar et al. (2024) suggested a mechanical interdependence between right heart function and respiratory function, explaining it by the fact that right ventricular (RV) stiffness leads to reduced lung volume and decreased left ventricular filling in patients with pulmonary arterial hypertension (PAH). All these findings were demonstrated using hemodynamic and imaging data [23]. The conclusions of this study provide a solid pathophysiological explanation for the reduction in lung volume observed in patients with insulin resistance (IR), mediated through right ventricular dysfunction (RVD).

In another study led by Boucherat et al. (2022), the pro-fibrotic protein LTBP-2 was identified and proposed as an early serum biomarker of right ventricular dysfunction, thereby demonstrating the connection between chronic inflammation, fibrosis, and cardiac decompensation [21].

The correlation between elevated IL-6 levels and impaired RV function was highlighted in the study by Prins et al., 2017, which demonstrated the role of the inflammation–fibrosis axis (via the JAK/STAT pathway) in the pathogenesis of RVD among patients with IR and PAH [18]. However, the data presented in this study are not directly applicable to diabetic patients, as these biomarkers have not yet been validated in longitudinal studies involving diabetic populations.

## 5. Therapeutic Management

The therapeutic approach for patients with type 2 diabetes (T2DM) and right ventricular dysfunction (RVD) involves strategies aimed at improving insulin sensitivity and protecting cardiac function. The interventions include the following:

- Lifestyle modifications: A balanced diet and regular physical activity can enhance insulin sensitivity and cardiovascular function. Weight loss has shown multiple benefits, including improvement of systolic ventricular function even in patients with severe heart failure. Additionally, it has demonstrated positive effects on right atrial and right ventricular systolic function [24,25].
- Pharmacological therapy: The use of medications that improve insulin sensitivity, such as metformin, and those that protect cardiac function, such as sodium–glucose Cotransporter-2 (SGLT2) inhibitors, can be beneficial [26,27].

Modern therapies used in the treatment of obesity, such as dual Glucagon-like peptide-1/GLP-1 and Gastric inhibitory polypeptide/GIP analogs (tirzepatide), have been shown to reduce the severity of obstructive sleep apnea symptoms, thereby indirectly improving respiratory function in these patients [28].

The use of this medication has been associated with a reduction in the apnea–hypopnea index (AHI), decreased reliance on noninvasive positive airway pressure devices (CPAP/PAP), and fewer hypoxemic episodes—all of which are consequences of weight loss.

Additionally, this medication has demonstrated benefits in patients with heart failure with preserved ejection fraction, suggesting potential improvements in right ventricular diastolic dysfunction and, as a result, lung volume [29].

A recent article discussed the benefits of using SGLT-2 inhibitors, e.g., Jardiance, in a patient at very high cardiovascular risk, with a history of cerebral artery aneurysm and heart failure. This class of medication has well-documented cardioprotective effects and shows therapeutic promise across a broad range of cardiovascular conditions [30].

A future research direction could be to evaluate whether this medication class also offers symptom relief in right-sided heart dysfunction and contributes to improved respiratory function.

- Monitoring of cardiac function: Regular evaluation of right ventricular function and respiratory parameters is essential for adjusting treatment and preventing complications.

Today, there are multiple methods for assessing cardiac function. In diabetic patients, cardiac evaluation is crucial given the well-established cardiovascular risk posed by diabetes—especially through macrovascular complications (e.g., myocardial infarction, heart failure) as well as microvascular complications such as cardiac autonomic neuropathy (CAN) [31]. One of the modern methods for detecting CAN in diabetic patients is the Sudoscan, which has proven valuable in the early detection and management of cardiovascular complications [32].

For the moment, no tailored treatment management is set in place for patients with RVD and insulin resistance; in future, more extensive research is needed in order to better understand the potential mechanism of the newer drugs and all the effects that they might have on these patients.

Currently, clinical research directions indirectly target the IR–RVD relationship. The SUMMIT study (Packer et al., 2025) showed that tirzepatide may have beneficial effects on improving right ventricular diastolic function and pulmonary capacity in patients with obesity and heart failure with preserved ejection fraction. These benefits were attributed to a reduction in adipose mass and a decrease in the apnea–hypopnea index (AHI) [29]. Although the results of this study are promising, it is important to note that it did not include diabetic patients with severe insulin resistance and did not directly assess lung volume.

Conversely, SGLT2 inhibitors (e.g., Jardiance) have demonstrated efficacy in reducing overall cardiovascular risk and appear to have potential benefits on right ventricular dysfunction as well. However, the available data are still limited to exploratory analyses [30].

Another indirect method for the early detection of RVD in diabetic patients is the monitoring of cardiac autonomic neuropathy (CAN) using techniques such as Sudoscan, though clinical validation is still ongoing [32].

Additional future studies are needed to evaluate the effects of tirzepatide and SGLT2 inhibitors not only on metabolic or hemodynamic parameters, but also on lung volume, right ventricular function, and inflammatory/fibrotic biomarkers, as shown in Table 1.

**Table 1:** Summary table of key studies on insulin resistance, right ventricular dysfunction, and pulmonary volume.

Author (Year)	Study Type	Population	Key Findings	Limitations	Reference No.
Min et al. (2022)	Observational cohort study	Subjects with IR and no cardiovascular disease	IR associated with systolic and diastolic RV dysfunction	No assessment of pulmonary function	[4]
Kang et al. (2019)	Experimental animal model	Diabetic rodents	IR leads to RV remodeling and contractile dysfunction	Limited human extrapolation	[5]
Zhang et al. (2024)	Cardiac MRI in HFrEF patients	Diabetic patients with heart failure	Diabetes worsens RVD in HFrEF patients	Cross-sectional design, cannot prove causality	[6]
Hemnes et al. (2019)	Observational study in PAH	PAH patients with IR	IR prevalent in PAH patients with highest mortality	No functional biomarker data	[13]
Brittain et al. (2016)	Cross-sectional study in PAH	PAH patients	Right ventricular lipotoxicity linked to fatty acid accumulation	No interventional insights	[12]
Boucherat et al. (2022)	Human proteomic studies	PAH patients with severe RVD	LTBP-2 identified as biomarker for RV failure	Needs prospective validation	[21]
Cubero Salazar et al. (2024)	Hemodynamic studies in PAH	PAH patients with low cardiac reserve	RV stiffness correlated with reduced lung volume	No healthy control group included	[23]

## 6. Implications: Why It Is Necessary to Study This Association and Its Potential Complications, Future Directions

Right ventricular dysfunction (RVD) has been shown to be an independent risk factor for increased mortality in patients with obesity and diabetes mellitus [11,33]. This elevated cumulative mortality risk provides a strong rationale for further investigating this association—especially given the dramatic rise in the global prevalence of both diabetes and obesity. Currently, there are approximately 830 million people worldwide living with diabetes [34], and as of 2022, more than 1 billion people were affected by obesity [35]. Worldwide, the prevalence of OSA is believed to be 1 billion persons affected by this condition, and the number is growing as the prevalence of obesity is also becoming higher [36].

Considering that the population of diabetic and obese patients continues to grow, and both conditions are strongly linked to increased cardiovascular risk—including the development of RVD—it becomes imperative to understand the underlying mechanisms of this association. Doing so will allow for the development of more targeted and individualized therapeutic strategies tailored to the patient’s specific needs.

In recent years, the global pandemic has forced us to rethink and reassess our approach to conditions previously acknowledged as severe but not fully appreciated in terms of their compounded impact under unfavorable circumstances. Elevated blood glucose levels in combination with pre-existing or newly developed cardiac pathologies have emerged as independent mortality risk factors in patients with diabetes, obesity, and impaired pulmonary function [37].

Moreover, hyperglycemia contributes to endothelial dysfunction through oxidative stress, creating a vulnerable environment for opportunistic pathogens such as fungi and bacteria. This was evident during the COVID-19 pandemic, where patients with elevated plasma glucose levels were more susceptible to opportunistic infections such as mucormycosis [38].

Considering the data presented above, we believe that future research directions should include:

- Prospective longitudinal studies to investigate the direct causal relationship between insulin resistance (IR), right ventricular dysfunction (RVD), and decreased respiratory volume;
- Evaluation of LTBP-2 and IL-6 as predictive biomarkers of RVD in populations with type 2 diabetes mellitus (T2DM) and severe IR [21];
- Randomized, double-blind, controlled clinical trials to assess the effects of tirzepatide and SGLT2 inhibitors on lung volume and right ventricular function in diabetic patients;
- Integration of CAN (cardiac autonomic neuropathy) assessment as a functional marker of early cardiac involvement in IR [32].

Such research could lead to the development of personalized screening and management protocols for patients with IR, T2DM, and RVD, ultimately reducing the risk of chronic respiratory failure and cardiovascular mortality.

## 7. Conclusions

Insulin resistance has a significant impact on right ventricular function in patients with type 2 diabetes mellitus, negatively affecting lung volume. Understanding the underlying mechanisms and implementing appropriate therapeutic strategies are essential for improving the prognosis and quality of life of these patients. Further studies are needed to understand the true clinical implications and potential complications resulting from this association, as well as to develop an adequate therapeutic strategy to support patients in this condition. Also, more focus needs to be held towards potential confounders such as concurrent cardiovascular diseases or other metabolic disorders that can affect diabetic patients and therefore can cause RVD in this patients.

## Data Availability

The original contributions presented in this study are included in the article. Further inquiries can be directed to the corresponding author.

## Abbreviations

The following abbreviations are used in this manuscript:

AHI	Apnea–hypopnea index
BMI	Body mass index
CAN	Cardiac autonomic neuropathy
CPAP/PAP	Noninvasive positive airway pressure devices
DCM	Dilated cardiomyopathy
GLP-1	Glucagon-like peptide-1
GIP	Gastric inhibitory polypeptide
HDL	High-density lipoprotein cholesterol
HR	Heart failure
IL-6	Interleukin 6
IR	Insulin resistance
IRS-1	Insulin receptor substrate-1
JAK/STAT	pathway to be the Janus kinase/signal transducers and activators of transcription
LTBP-2	Pro-fibrotic latent transforming growth factor-β binding protein 2
LDL	Low-density lipoprotein cholesterol
NT-proBNP	N-terminal pro b-type natriuretic peptide
OSA	Obstructive sleep apnea syndrome
PAH	Pulmonary arterial hypertension
PPAR-γ	Peroxisome proliferator-activated receptor gamma
RVD	Right ventricular dysfunction
RV	Right ventricle
SGLT2	Sodium-glucose Cotransporter-2 (SGLT2) Inhibitors
T2DM	Type 2 diabetes mellitus
